# Solitary submucous neurofibroma of the mandible: review of the literature and report of a rare case

**DOI:** 10.1186/1746-160X-5-24

**Published:** 2009-11-13

**Authors:** Rita Depprich, Daman D Singh, Petra Reinecke, Norbert R Kübler, Jörg Handschel

**Affiliations:** 1 Department for Cranio- and Maxillofacial Surgery, Heinrich-Heine-University, Moorenstr. 5, D-40225 Düsseldorf, Germany; 2 Department for Pathology, Heinrich-Heine-University, Moorenstr. 5, D-40225 Düsseldorf, Germany

## Abstract

Solitary neurofibroma is a rare benign non-odontogenic tumor. Particularly in the oral cavity, neurogenic tumors are rare, especially if they are malignant. Neurofibromas may present either as solitary lesions or as part of the generalised syndrome of neurofibromatosis or von Recklinghausen's disease of the skin. Clinically, oral neurofibromas usually appear as pediculated or sessile nodules, with slow growth and mostly without pain. The diagnosis can be confirmed by histological examination. Neurofibromas are immunopositive for the S-100 protein, indicating its neural origin. Treatment is surgical and the prognosis is excellent. For illustration a rare case of a solitary neurofibroma in the mandible is presented.

## Introduction

Neurogenic tumors are rare in the oral cavity, particularly so when malignant. Traumatic neuroma, although usually included with neurogenic tumors, is a reactive process rather than a true neoplasm [1]. In contrast, neurofibroma and schwannoma derive from nerve fibers, the perineurium, the endoneurium and the neurolemmomal cells [1]. Neurofibromas may present either as solitary lesions or as part of the generalised syndrome of neurofibromatosis or von Recklinghausen's disease of the skin [4-8]. Since the first description of solitary neurofibroma (neurilemmoma, schwannoma) of the oral cavity in 1954 by Bruce only few cases have been reported in the literature [2].

## Epidemiology

Although neurofibroma represents one of the most common neurogenic tumors it is an uncommon intraoral tumor [3] like some other intraoral tumours [4]. Neurofibromas can be multiple or solitary. The tumor's most frequent location is the skin and its multiple appearance is highly associated with von Recklinghausen's disease and poliglandular syndrome MEN III [5-9]. It mainly appears in the third decade of life although occurrence between 10 months and 70 years old has been described. Any preference of sex is reported contradictorily [6]. There are no correlations reported with immunocompromising diseases [10].

## Clinical features

Clinically, oral neurofibromas usually appear as pediculated or sessile nodule, with slow growth. They are usually painless, but pain or paresthesia may occur due to nervous compression. The most frequent location is the tongue, although they may occur at any site, especially on the palate, cheek mucosa and floor of the mouth [1,11-14]. Even intraosseous location of the mandible has been described [15-18]. The definitive diagnosis is due to histological examination.

## Pathohistological features

The macroscopic appearance of the oral neurofibroma is characterized by a whitish consistent mass with shiny surface. Microscopically the tumor is composed of an irregular pattern of proliferative spindle cells. The stroma is composed of collagen fibers and mucoid masses. Small axons all over the tumoral tissue are demonstrated with silver staining. Neurofibromas are immunopositive for the S-100 protein in 85 to 100% of the cases, indicating its neural origin [19-23].

## Treatment and prognosis

Treatment of choice is surgical excision of the solitary lesions, trying to conserve the nerve from which the tumor originates [5]. Malignant transformation of solitary neurofibroma is extremely rare. Recurrence is also rare although some authors suggest higher rate of recurrence at head and neck location of solitary neurofibromas [24-28]. Therefore, the prognosis is quite excellent.

## Case report

**Figure 1:**
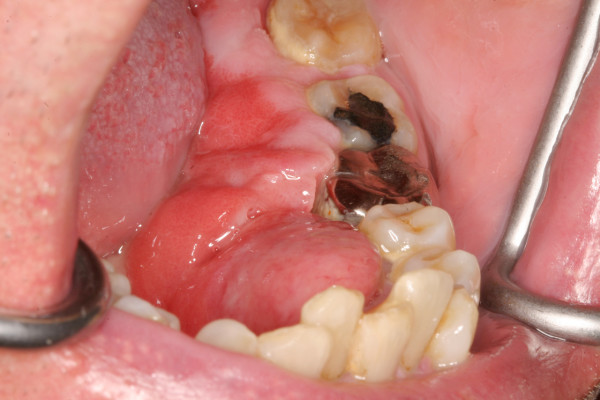
P**reoperative view: an exophytic tumor extending all along the lingual aspect of the left mandible**.

**Figure 2:**
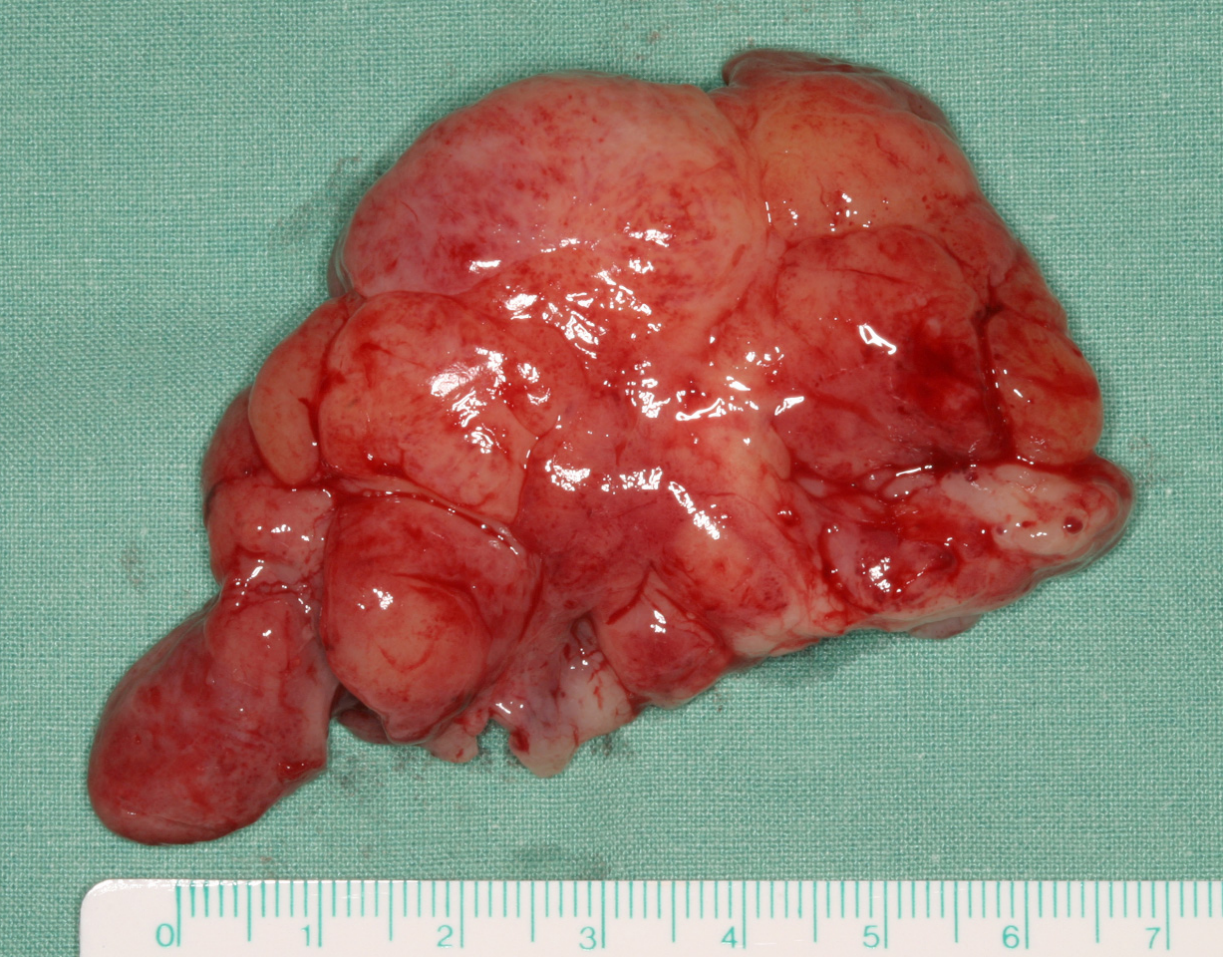
**Tumor mass after resection**.

**Figure 3:**
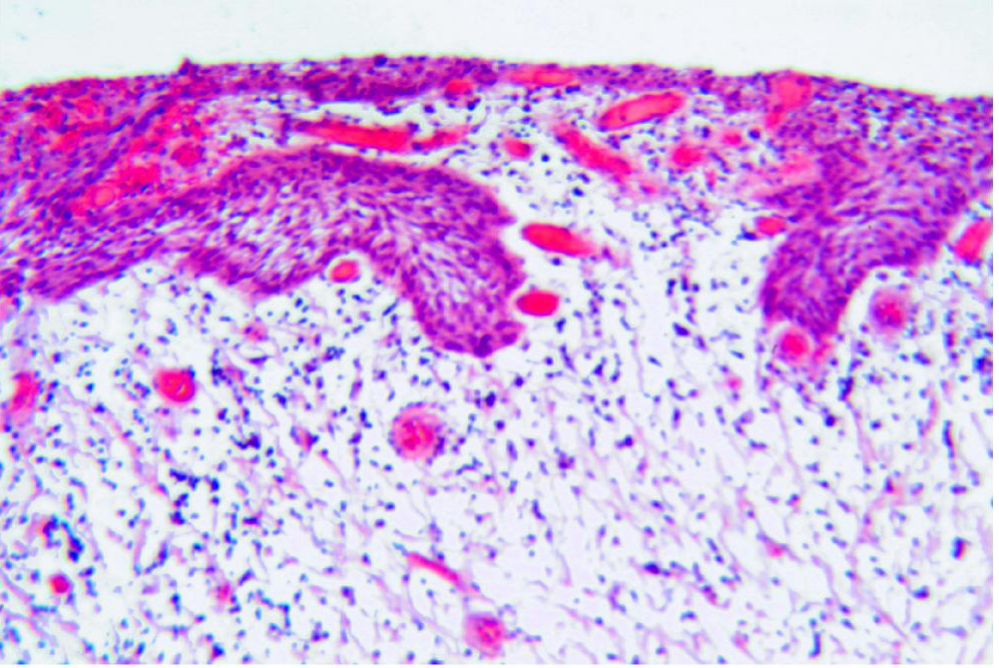
H**istological findings showing a tumor of proliferative spindle cells with a stroma composed of irregular collagen fibers (HE, × 100)**.

**Figure 4:**
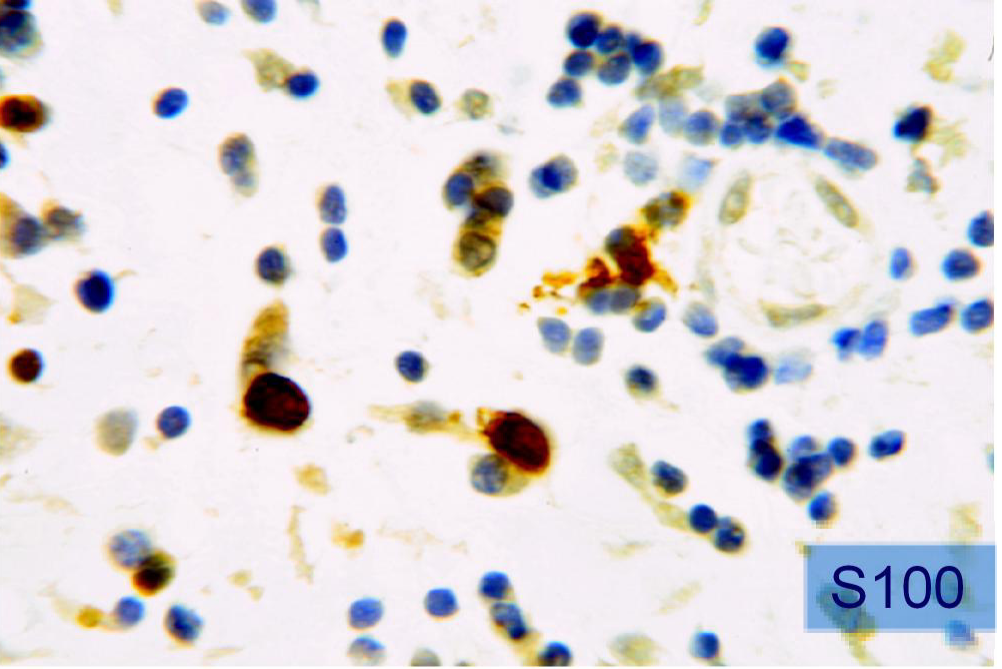
**Immunopositive staining for the S-100 protein (×320)**.

A 64-year-old male patient with a history of somewhat alcohol but no nicotine or any other diseases attended the department for Cranio- and Maxillofacial Surgery. Clinical examination revealed an exophytic tumor in the oral cavity extending all along the lingual aspect of the left mandible (fig. 1). Panoramic radiographs showed little to moderate interdental loss of bone between teeth 37 and 38 but no other abnormalities. Several biopsies from the oral cavity revealed a submucous benign mesenchymale proliferation with no signs of malignancy and thus, the tumor was completely excised under general anaesthesia (fig. 2, fig. 3). Surgical treatment also included extraction of teeth 37 and 38 and a modelling osteotomie. Immunohistochemical findings showed a solitary submucous neurofibroma with a predominate fibromatous component (fig. 4).

In the presenting case the lesion occured at the lingual site of the left mandible presenting as painless pediculated exophytic tumor with slow growth. The localisation supports the lingual nerve as origin for the neurofibroma. The patient presented no signs of von Recklinghausen disease or poliglandular syndrome. Preoperative panoramic radiographs showed no serious abnormalities. Definitive diagnosis was based upon histological and inmunohistochemical findings. A clinical follow-up has been regularly performed for one year, there was no evidence of recurrence until now.

## Competing interests

All authors disclaim any financial or non-financial interests or commercial associations that might pose or create a conflict of interest with information presented in this manuscript.

## Authors' contributions

DS, JH, RD and NK made substantial contribution to the conception and design of the manuscript. PR carried out the pathohistological investigations and participated in creating this part of the manuscript.

All authors were involved in revising the manuscript critically and have given final approval of the version to be published.

## Consent statement

Written informed consent was obtained from the patient for publication of this case report and accompanying images. A copy of the written consent is available for review by the Editor-in-Chief of this journal.

## References

[B1] Zachariades N Mezitis M Vairaktaris E Triantafyllou D Skoura-Kafoussia C Konsolaki-Agouridaki E Hadjiolou E Papavassiliou D Benign neurogenic tumors of the oral cavity Int J Oral Maxillofac Surg 1987 16 70 76 10.1016/S0901-5027(87)80033-4 3031179

[B2] Bruce KW Solitary neurofibroma (neurilemmoma, schwannoma) of the oral cavity Oral Surg Oral Med Oral Pathol 1954 7 1150 1159 10.1016/0030-4220(54)90307-2 13214714

[B3] Boedeker CC Ridder GJ Kayser G Schipper J Maier W Solitary neurofibroma of the maxillary sinus and pterygopalatine fossa Otolaryngol Head Neck Surg 2005 133 458 459 10.1016/j.otohns.2005.01.006 16143202

[B4] Handschel J Herbst H Brand B Meyer U Piffko J Intraoral sebaceous carcinoma Br J Oral Maxillofac Surg 2003 41 84 87 10.1016/S0266-4356(03)00036-6 12694699

[B5] Alatli C Oner B Unur M Erseven G Solitary plexiform neurofibroma of the oral cavity A case report Int J Oral Maxillofac Surg 1996 25 379 380 10.1016/S0901-5027(06)80036-6 8961022

[B6] Gomez-Oliveira G Fernandez-Alba Luengo J Martin-Sastre R Patino-Seijas B Lopez-Cedrun-Cembranos JL Plexiform neurofibroma of the cheek mucosa. A case report Med Oral 2004 9 263 267 15122129

[B7] Badger GR Solitary neurofibromatosis in the maxilla: report of oral findings J Am Dent Assoc 1980 100 213 214 6928152 10.14219/jada.archive.1980.0046

[B8] Che Z Nam W Park WS Kim HJ Cha IH Kim HS Yook JI Kim J Lee SH Intraosseous nerve sheath tumors in the jaws Yonsei Med J 2006 47 264 270 10.3349/ymj.2006.47.2.264 16642559 PMC2687639

[B9] Guneri EA Akoglu E Sutay S Ceryan K Sagol O Pabuccuoglu U Plexiform neurofibroma of the tongue: a case report of a child Turk J Pediatr 2006 48 155 158 16848118

[B10] Meyer U Kleinheinz J Handschel J Kruse-Losler B Weingart D Joos U Oral findings in three different groups of immunocompromised patients J Oral Pathol Med 2000 29 153 158 10.1034/j.1600-0714.2000.290402.x 10766392

[B11] Richards D Neurofibroma of the oral cavity Br J Oral Surg 1983 21 36 43 10.1016/0007-117X(83)90029-X 6404294

[B12] Shimoyama T Kato T Nasu D Kaneko T Horie N Ide F Solitary neurofibroma of the oral mucosa: a previously undescribed variant of neurofibroma J Oral Sci 2002 44 59 63 12058872 10.2334/josnusd.44.59

[B13] Sinha R Paul R Sen I Sikdar B A solitary huge neurofibroma of the soft palate J Laryngol Otol 2002 116 637 638 10.1258/00222150260171669 12389695

[B14] Skouteris CA Sotereanos GC Solitary neurofibroma of the maxilla: report of a case J Oral Maxillofac Surg 1988 46 701 705 10.1016/0278-2391(88)90117-6 3294358

[B15] Vivek N Manikandhan R James PC Rajeev R Solitary intraosseous neurofibroma of mandible Indian J Dent Res 2006 17 135 138 10.4103/0970-9290.29874 17176831

[B16] Ueda M Suzuki H Kaneda T Solitary intraosseous neurofibroma of the mandible: report of a case Nagoya J Med Sci 1993 55 97 101 8247113

[B17] Apostolidis C Anterriotis D Rapidis AD Angelopoulos AP Solitary intraosseous neurofibroma of the inferior alveolar nerve: report of a case J Oral Maxillofac Surg 2001 59 232 235 10.1053/joms.2001.20508 11213999

[B18] Sigillo R Rivera H Nikitakis NG Sauk JJ Neurofibromatosis type 1: a clinicopathological study of the orofacial manifestations in 6 pediatric patients Pediatr Dent 2002 24 575 580 12528952

[B19] Fisher DA Chu P McCalmont T Solitary plexiform neurofibroma is not pathognomonic of von Recklinghausen's neurofibromatosis: a report of a case Int J Dermatol 1997 36 439 442 9248889 10.1111/j.1365-4362.1997.tb01125.x

[B20] Johnson MD Glick AD Davis BW Immunohistochemical evaluation of Leu-7, myelin basic-protein, S100-protein, glial-fibrillary acidic-protein, and LN3 immunoreactivity in nerve sheath tumors and sarcomas Arch Pathol Lab Med 1988 112 155 160 2447857

[B21] Weiss SW Langloss JM Enzinger FM Value of S-100 protein in the diagnosis of soft tissue tumors with particular reference to benign and malignant Schwann cell tumors Lab Invest 1983 49 299 308 6310227

[B22] Depprich R Handschel J Sebald W Kubler NR Wurzler KK [Comparison of the osteogenic activity of bone morphogenetic protein (BMP) mutants] Mund Kiefer Gesichtschir 2005 9 363 368 10.1007/s10006-005-0644-2 16170576

[B23] Handschel JG Depprich RA Dirksen D Runte C Zimmermann A Kubler NR A prospective comparison of octyl-2-cyanoacrylate and suture in standardized facial wounds Int J Oral Maxillofac Surg 2006 35 318 323 10.1016/j.ijom.2005.10.003 16364595

[B24] Wise JB Patel SG Shah JP Management issues in massive pediatric facial plexiform neurofibroma with neurofibromatosis type 1 Head Neck 2002 24 207 211 10.1002/hed.10001 11891951

[B25] Polak M Polak G Brocheriou C Vigneul J Solitary neurofibroma of the mandible: case report and review of the literature J Oral Maxillofac Surg 1989 47 65 68 10.1016/0278-2391(89)90127-4 2642961

[B26] Takahama A Jr Leon JE de Almeida OP Kowalski LP Nonlymphoid mesenchymal tumors of the parotid gland Oral Oncol 2008 44 970 974 10.1016/j.oraloncology.2007.12.009 18282791

[B27] Becelli R Renzi G Cerulli G Saltarel A Perugini M Von Recklinghausen neurofibromatosis with palatal localization. Diagnostic and surgical problems in two clinical cases Minerva Stomatol 2002 51 391 397 12473976

[B28] Meyer U Wiesmann HP Berr K Kubler NR Handschel J Cell-based bone reconstruction therapies-principles of clinical approaches Int J Oral Maxillofac Implants 2006 21 899 906 17190299

